# A curious adult case of small bowel volvulus with congenital malrotation

**DOI:** 10.1093/jscr/rjac285

**Published:** 2022-06-15

**Authors:** Hao Han Tan, Goutham Sivasuthan, Man-Shun Wong

**Affiliations:** Department of General Surgery, Ipswich Hospital, QLD, Australia; Department of General Surgery, Ipswich Hospital, QLD, Australia; Department of General Surgery, Ipswich Hospital, QLD, Australia

## Abstract

Intestinal volvulus is defined as a twisting of the bowel on its mesentery. It itself is a rare occurrence, with documented incidence of 1% as the cause of all small bowel obstruction, with further 0.82% of them being associated with intestinal malrotation. The classical radiographic feature described in literatures is the whirlpool sign. We herein report a rare presentation of congenital malrotation causing a small bowel obstruction in a 43-year-old man. The patient presented with acute abdominal pain and underwent an emergency laparotomy and resection of small and large bowel (total of 3 m with primary anastomosis), with an estimated 2.6 m of viable small bowel left. The patient had a prolonged recovery complicated by another relook operation, superior mesenteric vein thrombus and a high-output stoma with subsequent electrolyte derangements and acute kidney injury. He was discharged on Day 26 and had been seen in the outpatient department with good functionality.

## INTRODUCTION

Intestinal volvulus is a rare but life-threatening condition that is defined as a twisting of the bowel on its mesentery resulting in a bowel obstruction. It requires timely diagnosis and urgent management due to its obstructive pathophysiology resulting in possible bowel infarction, perforation and sepsis. Small bowel volvulus is thought to be more common in the paediatric population due to the higher incidence of congenital associations (e.g. congenital malrotation, Merkel’s diverticulum) [1, 2]. It is even more rare in the adult population with limited studies suggesting an annual incidence of 1.7–5.7 per 100 000 adults in Western countries to 24–60 per 100 000 adults in Asian countries [3, 4].

This case highlights the rarity of these diseases and the importance of having them as part of the differential diagnosis for all patients with small bowel obstruction. It also highlights the classical clinical and radiological findings of small bowel volvulus, as well as the morbidities associated with it.

## CASE REPORT

**Figure 1:**
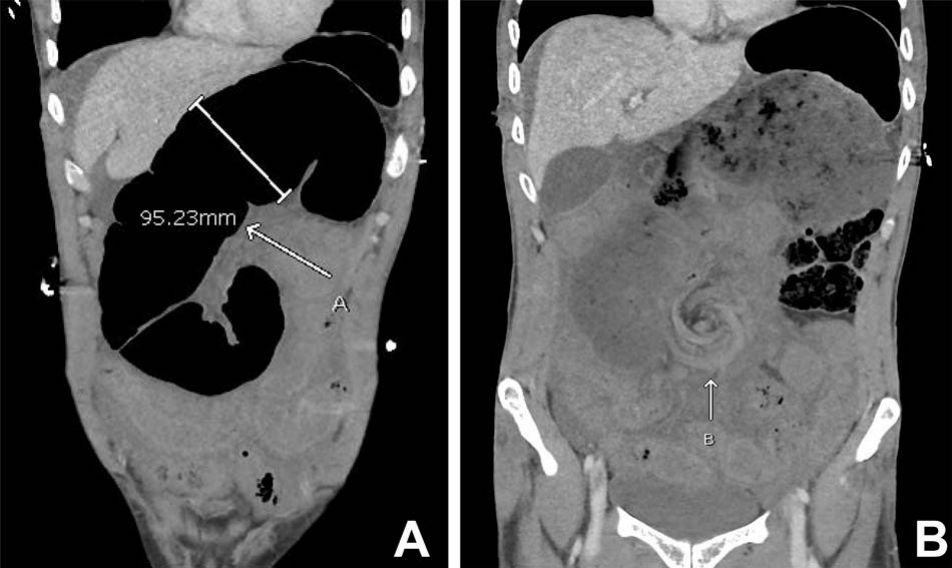
Initial CT abdomen pelvis with contrast in the portal venous phase showed (**A**) dilated small bowel and the (**B**) classical whirlpool sign.

A 43-year-old man presented with acute abdominal pain, nausea and vomiting in the context of not opening his bowels for 10 days. Otherwise, he was a fit and healthy gentleman with no previous abdominal surgeries. He was a light-smoker and did not consume any alcohol or illicit drugs. On presentation, the patient was unwell with diaphoresis and had a rigid abdomen with global peritonism. Rectal examination revealed no blood or mass. Blood tests showed a raised white cell count (27.8/L), borderline elevation of lactate (2.9 mmol/L) and normal C-reactive protein (< 2.0 mg/L). Computed tomography (CT) demonstrated small bowel obstruction with multiple dilated loops with twirling of the mesenteric vessels in central abdomen, as well as moderate ascites (Fig. 1).

The patient underwent an emergency laparotomy and resection of small and large bowel. He was found to have malrotation of the small bowel around its mesentery with associated haemoperitoneum. Given intra-op finding of non-viable intestine, a total of 3 m of intestine (2.6 m of small intestine +0.4 m of large intestine including appendix up to the transverse colon) was resected with primary anastomosis. An estimated 2.6 m of viable small bowel was left.

He was admitted to intensive care unit (ICU) post-operation given ongoing shocked state requiring two vasopressor agents. Day 2 post-operation, the patient was found to have small bowel herniating through his vacuum-assisted closure dressing, necessitating a re-look laparotomy. Given the ongoing shocked state, the decision was made to form an end-ileostomy and mucus fistula from transverse colon. The patient’s fascia was subsequently closed.

**Figure 2:**
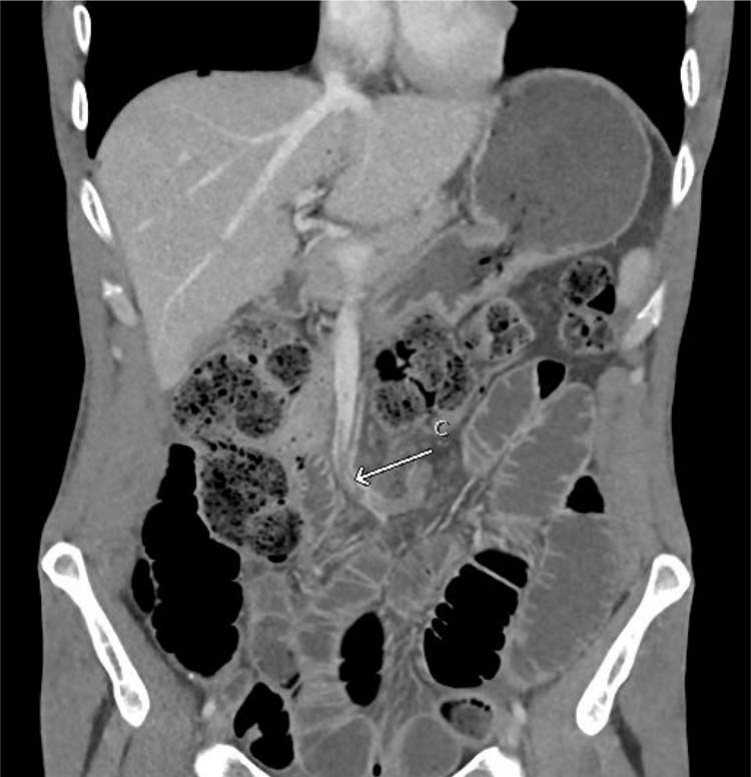
Repeat CT abdomen pelvis with contrast in the portal venous phase showed (C) filling defect in the superior mesenteric vein. Multiple dilated small bowel loops without a clear transition point were also evident, suggestive of ileus.

His admission was further complicated by acute kidney injury (AKI) secondary to high nasogastric + stoma outputs (6.6 L/day at peak) requiring aggressive fluid replacement and Loperamide + Metamucil. This was likely contributed by the relatively proximal nature of the stoma. Repeat CT also showed a thrombus within the left-sided branches of the superior mesenteric vein continuing into the vein proper (Fig. 2), which was managed as a provoked venous thromboembolism event with 6 months of therapeutic anticoagulation. Given prolonged malnutrition, a peripherally inserted central catheter was also inserted for total parenteral nutrition. Patient was discharged from ICU on Day 5 and had a total of 27 days in hospital with a normalized stoma output, resolved AKI and education to facilitate community management of stoma.

## DISCUSSION

Small intestine, or midgut, volvulus itself is a rare occurrence, especially in the adult population, with documented incidence of 1% as the cause of all patients presenting with small bowel obstruction. Further 0.82% of these patients were found to have associated intestinal malrotation as well [1]. Though the initial management of resuscitation is relatively standardized in obstructive surgical emergencies, this case emphasizes the importance of keeping a broad-based differential and careful peritoneal survey to understand the aetiology, which will help to guide intra-operative management.

This patient presented with clinically suspicious symptoms and an acute surgical abdomen, all of which were highly suggestive of an acute bowel obstruction. Patients with small bowel volvulus are more likely to present acutely with bowel ischaemia and peritonitis [2]. This was supported by the extensive bowel infarction found intra-operatively. Care must be taken in patients who present with more vague symptoms as they might present with intermittent obstruction, especially in the adult population.

This case also highlights the interpretation of the relevant investigations in the work-up of such patients. On admission, the patient’s lactate level (2.9 mmol/L) did not really correlate with his clinical status nor intraoperative findings. Although increased lactate is associated with strangulated bowel, the level can lag by 8 h [5]. In terms of imaging modality, CT is the most reliable with the classical whirlpool sign. Plain abdominal radiographs are not helpful in diagnosing the underlying cause, while ultrasonography is not as sensitive in the adult population [1].

In terms of management of small bowel volvulus, 65.2% of patients require some form of surgical intervention [3]. However, there is a wide variety in practices among surgeons. Although exploratory laparotomy is the mainstay therapy, laparoscopic surgery is becoming more popular. Likewise, if bowel resection is indicated, the decision for primary anastomosis versus ostomy is also variable, depending on clinical context. Given concerns for anastomosis breakdown due to ongoing shocked state in this patient, the surgical plan was modified to an end-ileostomy and mucus fistula.

This case also highlights the various morbidities and complications associated with the disease and its surgical interventions. Mesenteric venous thrombosis is a rare but reported complication after abdominal surgery, usually in the superior mesenteric vein, accounting for 1 in 1000 emergency surgical laparotomies for acute abdomen [6]. Malnutrition secondary to small bowel syndrome is another important morbidity of surgery, especially if <2 m of small intestine remains [7]. Other complications as evident in this case study include ileus, high nasogastric and stoma output.
